# Attitudes of Acutely Ill Patients Towards Euthanasia in Hong Kong

**DOI:** 10.2174/1874434600701010001

**Published:** 2007-07-26

**Authors:** R.C.S Lam, Wai-Tong Chien

**Affiliations:** 1 TM Hospital, Hospital Authority Hong Kong, Hong Kong SAR, P.R. China; 2 Nethersole School of Nursing, Faculty of Medicine, The Chinese University of Hong Kong, Shatin, N.T., Hong Kong SAR, P.R. China

**Keywords:** Euthanasia, patients’ attitude, acutely ill, Chinese.

## Abstract

The global euthanasia debate by health care professionals has raised important ethical issues concerning the professional duties and responsibilities of nurses caring for terminal patients. The purpose of this study was to examine the attitudes of acutely ill patients towards the practice of euthanasia in Hong Kong. A modified form of the 23-item Questionnaire for General Household Survey scale was used. This cross-sectional survey study was conducted with a stratified sample of in-patients recruited from a wide variety of departments in a regional, acute general hospital. Seventy-seven out of 129 patients responded (59.7%) and a high proportion of patients agreed with the use of euthanasia in the following circumstances: ‘where they were a third party’, if ‘someone they loved’ was affected, or if ‘they themselves were the patient’. Of the 77 patients, 54 agreed with active euthanasia (70.1%) and 65 with passive (84.4%). The results also indicated that a few socio-demographic characteristics (such as age, gender and household income) statistically significantly correlated with patients’ attitudes towards euthanasia. These findings highlight that Chinese patients with acute illness generally accept the use of euthanasia. Further research on the attitudes and perceptions of patients towards the use of euthanasia is recommended, particularly in diverse groups of Chinese and Asian patients with acute or terminal illness.

## INTRODUCTION

The world-wide euthanasia debate among health care professionals, sociologists and ethicists raises important moral issues. These life and death issues relate directly to the professional duties and responsibilities of nurses, as formal caregivers and advocates of patients.

Classically, euthanasia was defined as “the hastening of death of a patient to prevent further sufferings” [1]. This broad definition encompasses several terms used in connection with different forms of euthanasia, including: voluntary, involuntary and non-voluntary euthanasia; and active and passive euthanasia [2].

However, according to Chao, Chan and Chan [3], there is no real consensus as to the precise meaning of these terms. Voluntary euthanasia usually refers to euthanasia with the patient’s consent; where the patient has expressed a wish to die and another individual assists his/her death by performing the act of euthanasia. Chao *et al*. [3] explain that involuntary euthanasia does not involve the patient’s consent, even if the patient is competent to express his/her will and make decisions concerning the future, i.e. life saving or ending, but he/she has not been consulted and his/her life is ended by an act of euthanasia. Non-voluntary euthanasia refers to euthanasia performed when a patient is not competent to make decisions; e.g. when a patient is comatose, mentally insufficient, or unable to express his/her own wishes, i.e. babies born with severe congenital abnormalities.

The Code of Professional Conduct of Nurses [4] in Hong Kong and similar codes in Western countries such as the International Council of Nurses in Switzerland [5], state that a practicing nurse is obliged “to preserve human life by any necessary means and attempt to restore bodily functions”.

In 2001, laws, effectively decriminalizing acts of euthanasia, were passed in Belgium and The Netherlands. This provoked further debate among health professionals and ethicists, particularly with respect to the rights of and the benefits to patients and the need for an ethical stance on the professional duties of health professionals.

Nurses who work in acute and critical care settings often take care of patients with serious physical illnesses who may be near to death. They strive to provide the best treatment and to care for them. However, it is sometimes difficult to determine which treatment and care option would be most beneficial, and in the best interest of the patient, without understanding the patient’s wishes and perspective on their own care.

There has been limited research carried out and there is, therefore, limited evidence on the views of critically or terminally ill patients concerning continuing or withholding life support treatments. This study examined a randomized sample of acutely ill in-patients’ attitudes towards the practice of euthanasia.

The Hospital Authority of Hong Kong [6], under the definition of the Medical Council of Hong Kong, is firm in its stand against euthanasia. However, the Medical Council of Hong Kong [7] does not consider withholding artificial life support procedures for a terminally ill patient to be an act of euthanasia. This, according to Rachels [8] and other Western health care professionals, means that Hong Kong practices passive euthanasia. It is right that definitions of, and decisions concerning, euthanasia should be monitored and clarified by local government laws and regulations. However, in practice, doctors and other health professionals and patients themselves are usually the key people involved in the decision-making process and the implementation of euthanasia. Hence, they should inform policy and be involved in the legislative making process for euthanasia.

A few studies have been undertaken in the USA, the UK and Australia to identify the attitudes and beliefs of physicians and the general public concerning euthanasia. A quantitative study, conducted in the United States in 1993 [9], examined the attitudes of oncology patients and oncologists toward euthanasia and physician-assisted suicide. One hundred and fifty-five oncology patients and 355 oncologists were interviewed by telephone with vignette-style questions. About two-thirds of the patients found euthanasia acceptable for patients with unremitting pain. However, the patients found it harder to accept euthanasia and physician-assisted suicide in vignettes involving the themes of “burden on the family” and “life viewed as meaningless”. Patients in pain were more likely to find euthanasia or physician-assisted suicide ethically unacceptable and an inappropriate response to poor pain management. Patients with life-threatening illnesses often experienced associated depression and psychological distress and were significantly more likely to have seriously discussed euthanasia. Despite nearly one in seven oncologists having carried out euthanasia or physician-assisted suicide, the majority found euthanasia or physician-assisted suicide ethically unacceptable, even for patients with unremitting pain.

In addition, studies have shown that patients’ demographic and illness characteristics (such as age, gender, type and duration of illness and family financial situation) may affect their perceptions, the decisions made about treatment, and the use of euthanasia [9–11]. However, these studies did not use large samples of patients from different socio-demographic and cultural backgrounds or different illness conditions [3,11]. Information concerning the views of Chinese and Asian patients on the use of euthanasia is limited.

Mak and Elwyn [10] also conducted an investigation into the desire for euthanasia in advanced cancer patients in a hospice in Hong Kong. This qualitative study, where data was collected from six Chinese palliative care patients, indicated that patients’ covert “desire for euthanasia” was not really about dying, but about care, connectedness and respect. As a result, the interpretation of the meaning of euthanasia is an important issue in the care of terminally ill patients. Patients who request such an intervention should be assessed and evaluated carefully before euthanasia can be considered seriously.

Nevertheless, few of these previous studies deal with the attitude of patients in acute hospitals and very little research has been done to explore attitudes in Chinese and Asian populations. Since patients are the recipients of and potential decision makers about euthanasia, their views on the subject should be sought.

## AIMS OF THE STUDY

The aim of this study was to investigate the attitudes of patients towards euthanasia in an acute general hospital in Hong Kong.

The objectives of the study were:

- to examine the attitudes of patients towards the use of euthanasia in an acute general hospital in Hong Kong;
- to identify any correlation between the attitudes of patients and their socio-demographic characteristics and state of health; and
- to compare male and female patients’ attitudes towards euthanasia and to identify any differences between them.

## METHOD

In April 2004, a cross-sectional survey was conducted in a 2,000-bed regional, acute general hospital in the largest geographical region of Hong Kong. A stratified random sample of 129 patients was selected from the patient list (839 patients at the time of the study) using a computer-generated table of random numbers. The sampling strata, based on the accessible clinical specialities in the hospital, included patients from Medicine and Geriatrics (49), Surgery (26), Orthopaedics and Traumatology (18), Oncology (15), Obstetrics and Gynaecology (13) and Anaesthesia and Intensive Care (8). About 15% of the patients in these six departments were randomly and proportionately selected at subject recruitment, using a random number table. The sample only included patients over 18 years old who were free from psychiatric illness or cognitive impairment. This sample size allowed for a ± 0.05 sampling error with a 95% confidence level and a non-response rate of up to 45% [12]. A stratified sampling method was adopted to obtain an approximately equal proportion of eligible subjects from each of the clinical specialties in the hospital, and thus ensured that the final sample would represent the acutely ill patient population more effectively [13].

Permission to conduct the study was obtained from both the hospital and the University’s Clinical Research Ethics Committee. A research nurse checked patients’ background information in the wards against the study criteria. She then approached the patients (eligible subjects) and fully explained the purpose and procedure of this study to them. Written consent was sought from each patient to ensure that they were participating in the study on a voluntary basis. They were also assured of anonymity, confidentiality of personal data, and were given the right to withdraw from the study at any time.

After written consent was obtained, all patients completed a self-reported 23-item modified form of the Questionnaire for General Household Survey [11]. This was used to identify the attitudes of the patients (and hence the general public) towards euthanasia. They also completed a demographic data sheet supplying their age, gender, education level, medical diagnosis and household income.

With no specific theoretical framework in the topic of euthanasia, the questionnaire was developed and based on findings on the important issues linked with active and passive euthanasia in recent literature [11]. The questionnaire was designed for both doctors (Section 1) and the general public (Section 2) in Hong Kong, and the second section was used in this study. Feedback from an expert panel, (2 nurse specialists, 2 doctors, a ward manager, a medical patient, and a family carer) clarified the instructions for administration of the questionnaire, altered the register of the language for use in a hospital setting, and a few vignettes were amended accordingly. The modified form of the questionnaire demonstrated satisfactory internal consistency (Cronbach’s alpha= 0.82) in the pilot study in a suitable sample of 20 acutely ill patients in the hospital studied [14]. Made up of 3 parts, it asked about: (Part 1) attitudes towards euthanasia in general (15 items); (Part 2) attitudes towards passive euthanasia (4 items), using a scenario in which a patient requests the use of passive means to allow them to die; and (Part 3) attitudes towards active euthanasia (4 items) using a scenario similar to the one in Part 2, but in which a patient requests the use of active means to hasten their death. The responses were rated using a 5-point Likert-type scale (1- strongly disagree to 5- strongly agree), i.e. the higher the score, the higher the level of acceptance of euthanasia. The Cronbach’s alpha coefficients ranged from 0.77 - 0.83, indicating a satisfactory internal consistency [13].

Descriptive statistics, such as percentages and means, were used to summarize the results. To determine whether parametric statistical tests could be used, univariate normality of the item scores was checked. Despite the narrow range of the item scores, most of them were found to be distributed normally. Pearson’s correlation test was used to examine the relationships between the scores given by the patients and their age, income and length of hospital stay (interval data). Spearman’s correlation test was employed to test the relationships between the scores given by the patients and their other characteristics, such as their education level and any co-morbidity of other diseases (ordinal data). A two-tailed paired sample t-test was used to compare any difference between the patients’ attitudes towards passive and active euthanasia. An independent samples t-test (two-tailed) was used to compare any difference in the attitude towards euthanasia between male and female patients. The statistical significance of all tests was set at 0.05.

## RESULTS

Seventy-seven questionnaires were returned, giving a response rate of 59.7%. The mean age of the patients was 49.8 years old (SD = 16.8; range 20 - 73 years). A similar number of patients was recruited: 40 male (51.9%) and 37 female (48.1%). Less than half (42.0%) had a religious belief, mainly Christian, Protestant (22.0%) or ancestor worship (20.0%). More than three-quarters (78.8%) were educated to secondary school or university level. About half (48.5%) had a monthly income between Hong Kong $ 10,001 and $ 30,000 (US$ 1,280–$ 3,845). The respondents included: 32 patients from Medicine and Geriatrics (41.6%), 15 from Surgery (19.5%), 10 from Orthopaedic and Traumatology (13.0%), nine from Oncology (11.7%), seven from Obstetrics and Gynaecology (9.1%) and four from Anaesthesia and Intensive Care unit (5.2%).

In this study, the Cronbach’s alpha coefficients of the three parts of the questionnaire ranged from 0.79 - 0.86, indicating a satisfactory internal consistency. The mean score of the general attitude towards euthanasia (Part 1) was 4.05 (SD = 0.59). The items with the five highest mean scores amongst the 15 items in Part 1, and the item means and standard deviations indicating the attitudes of the patients towards passive and active euthanasia, are summarized in Table **1**. Most of the patients accepted the use of active (70.1%) and passive (84.4%) euthanasia in all three of the following circumstances: in cases where they were a third party, in the case of someone they loved, or in the case that they themselves were the patient (i.e. all mean values >3.00). However, they indicated statistically significantly more acceptance of passive euthanasia (M = 3.97 – 4.08), than of active euthanasia (M = 3.00 – 3.35), with the t values ranging from 5.17 to 6.12 and all p values were < 0.005.

The results of the correlation tests indicated that the mean score on the attitude of the patients correlated statistically significantly with age, gender and household income. The patients who had a lower monthly income (Pearson’s r = 0.35, p = 0.01) and were older in age (r = -0.40, p = 0.005) showed a higher acceptance (i.e. a higher attitude score) of euthanasia. The results of the unpaired t-test indicated that more female than male patients supported the use of euthanasia (t = 3.90, p = 0.04). Otherwise, there was no statistically significant correlation between the attitude score and other patient characteristics.

## DISCUSSION

The high mean scores (> 3.00) on attitudes towards passive and active euthanasia indicate that most patients agreed with the use of euthanasia (i.e. ranged from 3 - somewhat agree to 5 – strongly agree).

Respondents agreed that ‘a patient with terminal illness has the right to decide to die’ and accepted the idea of ‘withholding or withdrawal of life-sustaining treatment in order to let a terminally ill person die’. They would support and respect such requests from a patient, even if the person in question were someone they loved, or if they themselves were the patient. However, fewer respondents expressed acceptance of the use of active means of euthanasia, such as the application of treatment to hasten a patient’s death, rather than passive means of euthanasia.

The findings reflect the attitudes of poorer and older people who may consider that their illness and hospitalisation places a heavy burden and strain on their whole family, in financial, psychological, social and physical terms. Similar results have been found among the general population in Hong Kong, as reported by Fok *et al*. [11]. As the concept of ‘not being a family burden’ is common in Chinese culture, euthanasia may be considered by some critically ill patients to be an acceptable means of ending, not only their own suffering, but that of their family as well.

In addition, the findings indicate that female patients have a higher acceptance of euthanasia than male patients. This may be explained by the fact that women in Chinese society, particularly the older generation, are seen to be responsible for caring for other family members. They would blame themselves, therefore, if they did not take responsibility for their own death, thus releasing other family members from having to worry about their illness or state of health [15]. This traditional gender role in a Chinese family might contribute to the female patients being more accepting of euthanasia than their male counterparts.

Passive euthanasia was generally more acceptable than active euthanasia to the patients in this study. Emotionally and morally, Chinese patients are more receptive to the idea of a ‘natural death’ than to the idea of life-ending interventions, such as injections to hasten death [16]. This finding is also consistent with Western studies on attitudes towards physician-assisted suicide. However, Chinese patients’ perceptions of passive euthanasia and its related personal, cultural and illness issues should be explored in future research.

The findings of this study identified that acutely ill patients expressed a preference and desire for the use of euthanasia and they generally accepted the practice of euthanasia, especially passive euthanasia. This highlights the necessity for nurses to ensure that patients have a means of expressing their desire for death with dignity, to reduce suffering from critical or terminal illness. The Code of Professional Conduct and Code of Ethics for Nurses [4] suggests that this is one of the nurses’ professional duties, thus nurses can safeguard informed decision making of patients about the treatment and care provided for them [17,18], and ensure that decisions made by doctors, relatives, or patients, to withhold or withdraw treatment would be in the best interest or to the benefit of the patient concerned.

In addition, more information and understanding about patients’ attitudes and values towards euthanasia can improve communication between patients, doctors, nurses, allied health care staff and relatives regarding the treatment alternatives, or a ‘Do-Not-Resuscitate’ decision requested by the patient him/herself [15]. The findings on the patients’ attitudes towards euthanasia in this study also highlight that nurses should act as advocates for their patients whenever necessary, expressing the patient’s wishes and requests for the use of euthanasia. Knowledge of patients’ attitudes towards euthanasia and the factors influencing their attitudes would also be useful for nurse managers, physicians and hospital administrators when designing or reviewing the policies of life support treatment and decisions on resuscitation for critically or terminally ill patients.

Euthanasia is an important but controversial topic in hospital services and the findings of this study did not identify the ethical issues surrounding decisions and acts of euthanasia. Subjective biases, or the Hawthorne effect, may have been introduced by the circumstances such as the patients’ health and/or the ward environment in which the patients’ attitudes were assessed by the research nurse. This study examined the attitudes of patients, however, the perspectives on euthanasia from family members, medical and nursing staff should be sought in order to get a comprehensive view of the topic concerned. Future research should try to address these limitations.

## CONCLUSION

In spite of only a small sample being recruited in one general hospital, the findings of this study suggest that Chinese in-patients with acute illness generally accept the use of active and passive euthanasia. Socio-demographic factors identified in this study, such as age and monthly household income, may affect patients’ attitudes towards the practice of euthanasia. Female patients indicated a higher acceptance of euthanasia than male patients and this may be due to their caring gender role in the Chinese family. Further research on this topic is recommended, using larger samples of Chinese and other Asian patients with different types of critical or terminal illness.

## Figures and Tables

**Table 1:** Selected Results of Questionnaire on Attitudes Towards Euthanasia (N = 77)

	Range	M	SD
**General attitude towards euthanasia (total score)** **Five items with the highest mean scores:** To me, euthanasia should be accepted in today’s society. A person with a terminal illness has the right to decide to die. Euthanasia is helpful at the right time and place (under the right circumstances). Euthanasia is acceptable in cases when all hope of recovery is gone. If a terminally ill or injured person is increasingly concerned about the burden that his deterioration of health has placed on his family, I will support his request for euthanasia.	1 - 5 1 - 5 1 - 5 2 - 5 2 - 5 1 - 5	4.05 4.50 4.41 4.37 4.25 4.20	0.59 0.65 0.61 0.68 0.56 0.62
**In order to be relieved of pains and sufferings, a terminally ill person makes a personal request for the use of PASSIVE MEANS, such as the withdrawal of life-sustaining treatments, to let him/her die.** I will support this person’s request. If this person were someone I loved, I would support him/her to make such a request. If I were this person, I would make this request.	2 - 5 1 - 5 1 - 5	4.02 3.97 4.08	0.63 0.53 0.42
**In order to be relieved of pains and sufferings, a terminally ill person makes a personal request for the use of ACTIVE MEANS, such as application of treatment to hasten his/her death.** I will support this person’s request. If this person were someone I loved, I would support him/her to make such a request. If I were this person, I would make this request.	1 - 5 1 - 5 1 - 5	3.35 3.22 3.00	0.60 0.62 0.52

Note: Scores range from 1 – ‘strongly disagree’ to 5 – ‘strongly agree’. M, mean; SD, standard deviation.

## References

[1] Backer BA, Hannon N, Russell NA (1994). Death and dying: understanding and care.

[2] Hunt T, Penson J, Fisher R (1995). Ethical issues. Palliative care for people with care.

[3] Chao DVK, Chan NY, Chan WY (2002). Euthanasia revisited. Fam Pract.

[4] Nursing Council of Hong Kong (2002). Code of Professional Conduct and Code of Ethics for Nurses in Hong Kong.

[5] International Council of Nurses (2003). Biennial Report 2002-2003.Geneva, Switzerland: ICN.

[6] Hospital Authority of Hong Kong (2002). HA guidelines on life-sustaining treatment in the terminally ill.

[7] Medical Council of Hong Kong (2000). Processional code and conduct for the guidance of registered medical practitioners.

[8] Rachels J, Baird RM, Rosenbaum SE (1989). More impertinent distinctions. Euthanasia: the moral issues.

[9] Emanuel EJ, Fairclough DL, Daniels ER, Clarridge BR (1996). Euthanasia and physician-assisted suicide: attitudes and experiences of oncology patients, oncologists, and the public. Lancet.

[10] Mak Y, Elwyn G (2000). The meaning of desire for euthanasia in advanced cancer patients. Unpublished Master’s thesis.Swansea, UK:University of Wales Swansea.

[11] Fok SY, Chong MLA, Tang KC (2000). Public and doctors’ attitudes toward euthanasia in Hong Kong.

[12] Wang MQ, Fitzhugh E, Westerfield RC (1995). Determining sample size for simple-random surveys. Health Values.

[13] Portney LG, Watkins MP (2000). Foundation of clinical research: application to practice.

[14] Lam, CSR (2004). A comparison between doctors and patients’ attitudes towards euthanasia in an acute general hospital in Hong Kong. Unpublished Master’s dissertation, Nethersole School of Nursing, The Chinese University of Hong Kong.

[15] Leung KK, Chien WT, Mackenzie AE (2000). Needs of Chinese families of critically ill patients. Western J Nurs Res.

[16] Chien WT, Chiu YL, Lam LW, Ip WY (2006). Effects of a needs-based education programme for family carers with a relative in an intensive care unit: a quasi-experimental study. Int J Nurs Stud.

[17] Henderson A, Chien WT (2007). Health beliefs and expectations implicit in decision-making in a Hong Kong Chinese surgical population. J Clin Nurs.

[18] Chien WT, Norman I, Thompson DR (2006). Perceived benefits and difficulties experienced in a mutual support group for family carers of people with schizophrenia. Qual Health Res.

